# Recurrent secondary hyperparathyroidism after parathyroidectomy due to anterior mediastinum ectopic parathyroid glands in a peritoneal dialysis patient—a case report and literature review

**DOI:** 10.3389/fmed.2025.1564135

**Published:** 2025-06-16

**Authors:** Wen Cao, Wei Qing, Haoyuan Ren, Bin Song

**Affiliations:** ^1^Department of Nephrology, Deyang People’s Hospital, Deyang, China; ^2^Department of Gastrointestinal Surgery, Deyang People’s Hospital, Deyang, China

**Keywords:** peritoneal dialysis, ectopic parathyroid glands, parathyroidectomy, recurrent secondary hyperparathyroidism, hungry bone syndrome

## Abstract

**Background:**

Secondary hyperparathyroidism (SHPT) is a common complication of end-stage kidney disease (ESKD). Parathyroidectomy (PTX) is a reasonable option for patients with ESRD complicated by refractory SHPT, although only a few cases of ectopic parathyroid glands have been reported in patients undergoing peritoneal dialysis (PD).

**Case presentation:**

We present a case of recurrent SHPT after parathyroidectomy due to ectopic parathyroid glands in a patient undergoing long-term PD for approximately 10 years. The ectopic parathyroid glands were identified when performing the first operation, but it was too risky to be removed, and it induced recurrent SHPT. The reoperation was successful, aided by precise localization through Technetium-^99m^ methoxyisobutylisonitrile single photon emission computed tomography/computed tomography (^99m^Tc-MIBI SPECT/CT) imaging and advanced thoracoscopic surgical techniques. The patient received a calcium concentration of 1.75 mmol/L dialysate, combined with adequate calcium and active vitamin D supplementation, immediately after the second PTX, which resulted in the remission of hungry bone syndrome (HBS) within a short time.

**Conclusion:**

Accurate evaluation of the position and function of the parathyroid gland before PTX is the key to reducing the rates of missed diagnosis and SHPT recurrence. Integrated management based on mineral metabolism assessment is crucial for preventing and treating hungry bone syndrome after ectopic PTX in clinical practice.

## Introduction

Secondary hyperparathyroidism (SHPT) is a common complication in end-stage kidney disease (ESKD) and is a significant cause of renal osteodystrophy, anemia, calciphylaxis, ectopic calcifications, and cardiovascular mortality (1). Parathyroidectomy (PTX) is a recommended treatment for SHPT patients who fail regular drug therapy, including vitamin D analogues and calcimimetics. A successful PTX is associated with reduced risk of all-cause mortality and cardiovascular mortality in patients with ESKD (2). However, recurrent SHPT after PTX can occur in up to 32.14% of patients, regardless of the procedure type (3, 4). Ectopic parathyroid glands, which appear in 6.6–26% of renal hyperparathyroidism (HPT) cases (5, 6) and represent a significant cause of refractory HPT, play a crucial role in causing recurrent SHPT after PTX and need to be further emphasized in clinical practice. Compared to hemodialysis patients, the relatively smaller population size of peritoneal dialysis (PD) recipients has resulted in inadequate attention being paid to precision diagnosis, treatment, and personalized postoperative management strategies for ectopic parathyroid glands in this specific cohort.

Therefore, we report a case of recurrent secondary HPT after PTX due to an anterior mediastinal ectopic parathyroid gland in a patient receiving long-term PD. This case underscores the significance of accurate preoperative diagnosis and personalized postoperative management strategies for this population.

## Case presentation

**Figure 1:**
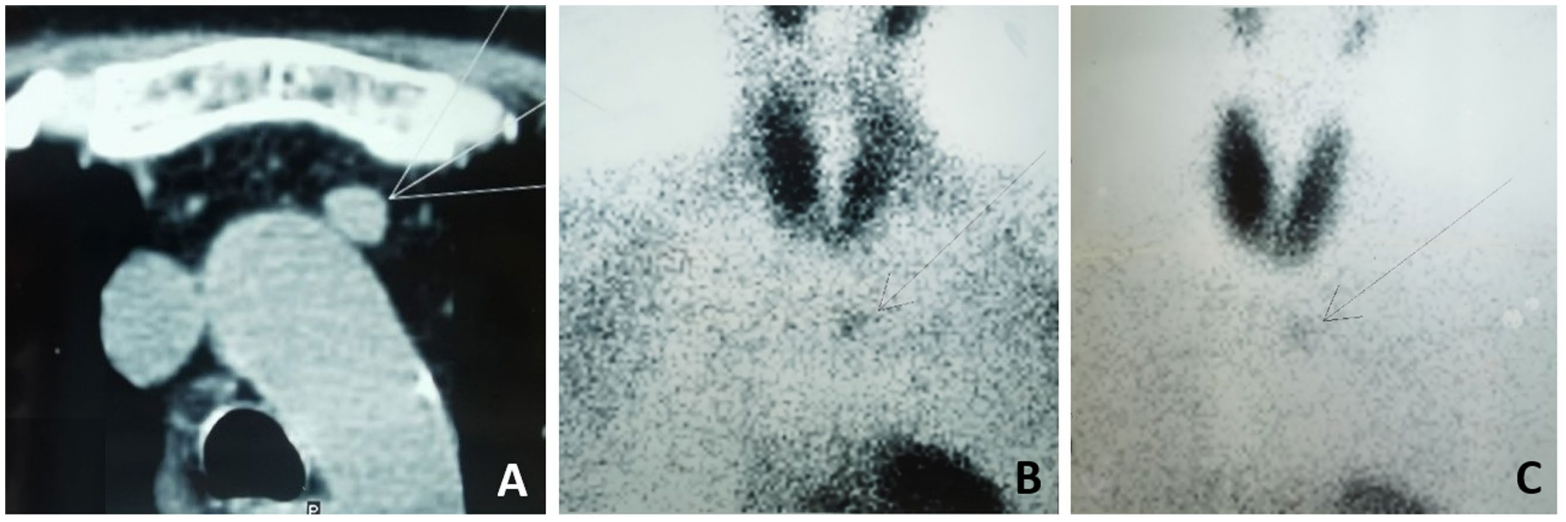
Chest CT scan and ^99m^Tc-MIBI before the 1st surgery. **(A)** Chest CT showed a 12 x11-mm nodule adjacent to aortic arch. **(B)** ^99m^Tc-MIBI showing increased uptake in the anterior mediastinum in the early phase after 30 minutes and **(C)** in the delay phase after 120minutes.

A 49-year-old woman with ESKD had been on PD for 11 years due to a 12-year history of nephrotic syndrome of unknown etiology. SHPT was well-controlled for approximately 6 years with treatment consisting of activated vitamin D agents and phosphate binders. In the seventh year after initiating PD, increases in serum intact parathyroid hormone (iPTH) levels, hypercalcemia, and hyperphosphatemia were observed. Activated vitamin D agents were discontinued and replaced with cinacalcet hydrochloride. However, the patient continued to experience progressive SHPT, with iPTH levels rising to 1,114 pg./mL in August 2019, accompanied by severe bone pain and skin itching, prompting her to seek further treatment at the local hospital. Technetium-99 m-sestamibi imaging (^99m^Tc-MIBI) revealed persistent focal uptake in the delayed phase at the dorsal upper and lower poles of the right thyroid lobe, the dorsal middle of the left thyroid lobe, and the anterior mediastinum (Figures 1B,C). Computed tomography (CT) further identified a 12 × 11-mm nodule adjacent to the aortic arch (Figure 1A). PTX was performed, and three ectopic parathyroid glands were removed in October 2019. The ectopic parathyroid gland in the anterior mediastinum was left intact for the following reasons: (i) its relatively weaker ^99m^Tc-MIBI uptake compared to the other glands indicated weaker function; and (ii) its location in the anterior mediastinum adjacent to the aortic arch posed a high surgical risk. After the surgical risks were explained to the patient, she expressed significant concern and hesitation and ultimately decided against lesion removal. The initial surgery appeared successful, as evidenced by a decrease in serum iPTH levels to 303 pg./mL. However, HPT recurred with a progressive increase in serum iPTH levels to 2,703 pg./mL within 3 years after the surgery, despite the patient’s continuous use of phosphate binders and cinacalcet hydrochloride.

**Figure 2:**
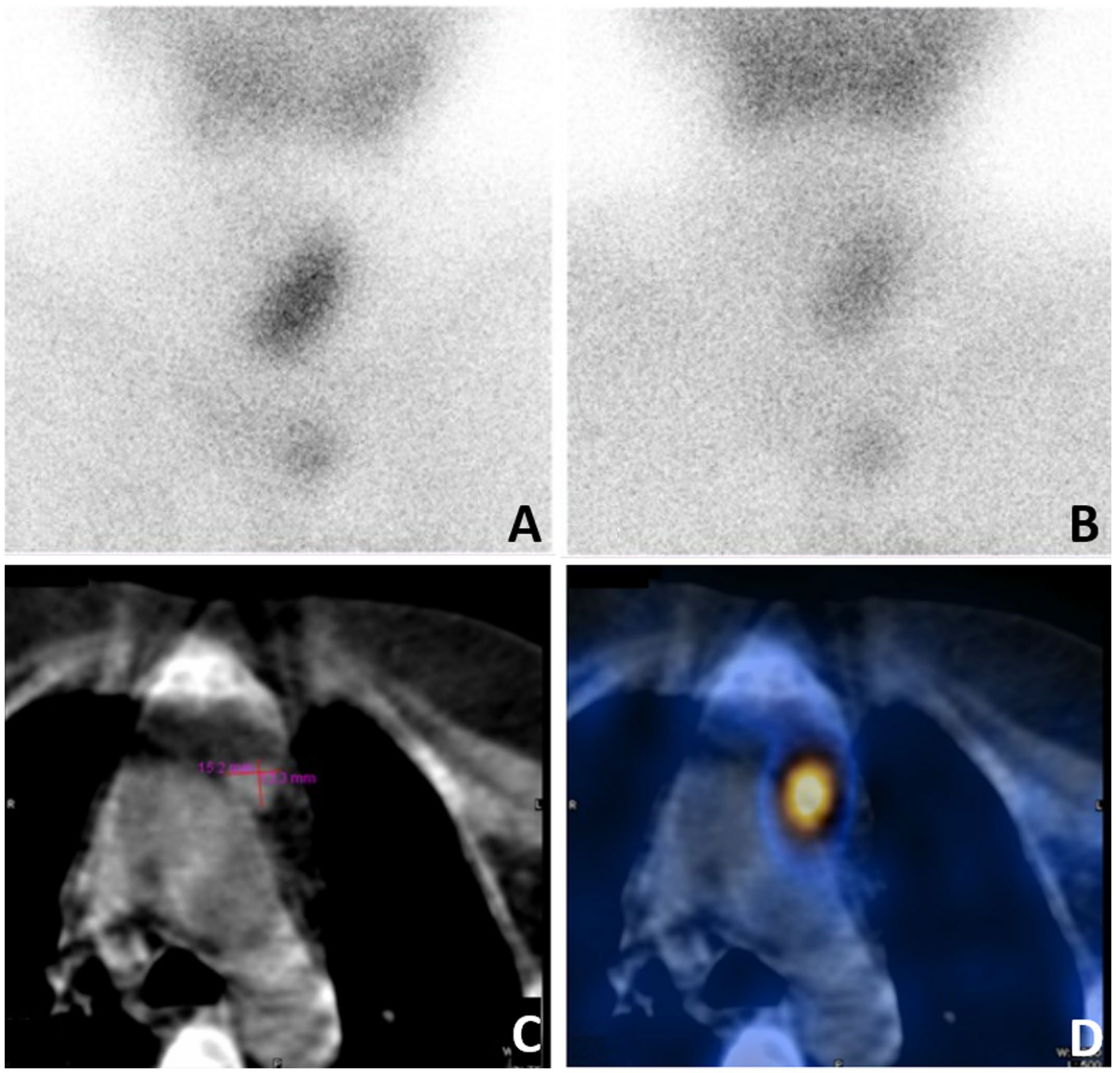
Reexamination of ^99m^Tc-MIBI and SPECT/CT before the second operation. **(A)** ^99m^Tc-Sestamibi scintigraphy demonstrating increased uptake on early acquisition, **(B)** subtle persistance on 2-hour delayed acquisition. **(C)** CT showed a 15.2 x13.3-mm nodule adjacent to aortic arch in anterior mediastinum. **(D)** SPECT/CT showed focal increased activity in anterior mediastinum.

**Figure 3:**
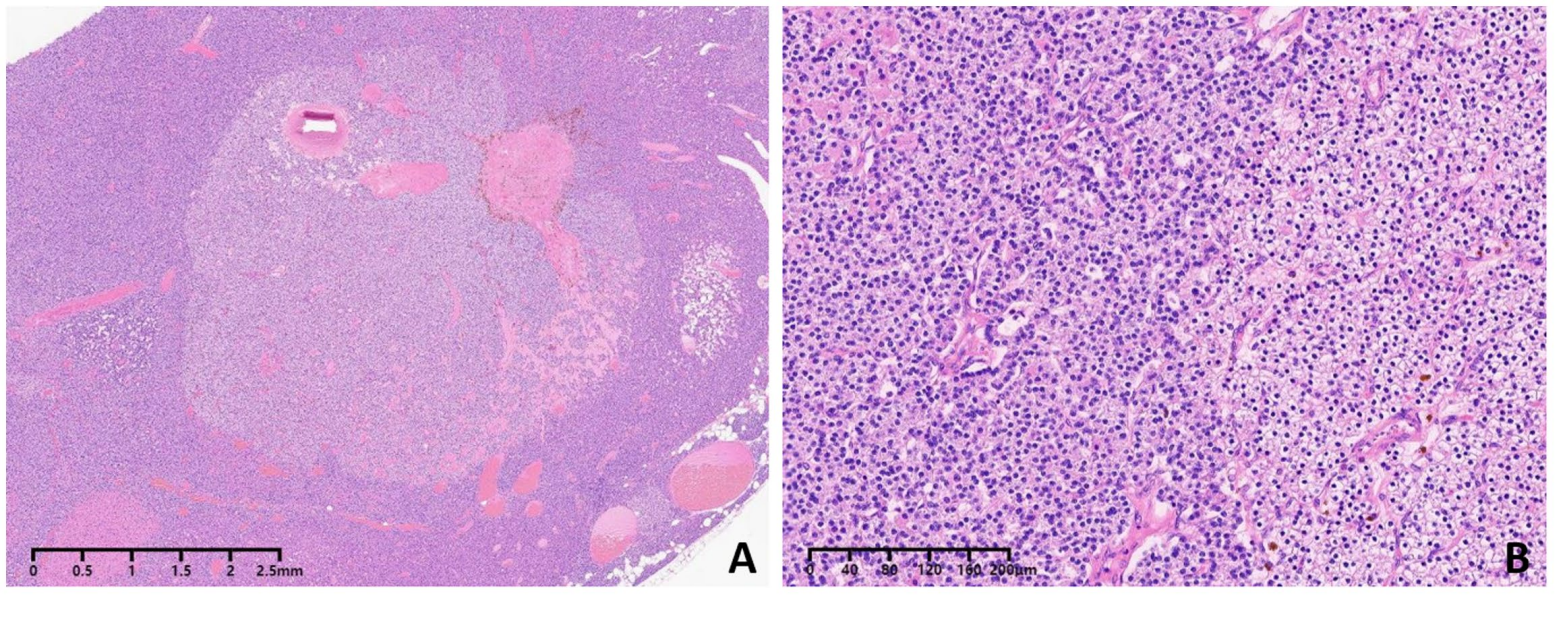
Pathologic analysis showed parathyroid adenoma with nodular parathyroid hyperplasia **(A,B)**. Hematoxylin and eosin staining (magnification, 40 for **A**, 400 for **B**).

Given this complex situation, the patient was referred to our hospital for a radical operation. Technetium-99 m-sestamibi (^99m^Tc-MIBI) and single-photon emission computed tomography (SPECT)/CT were used to re-evaluate the ectopic parathyroid gland, revealing an increase in size to 15.2 × 13.3 mm and a significant enhancement in ^99m^Tc-MIBI uptake (Figure 2). A comprehensive assessment of bone metabolism was conducted before the initiation of PTX: the bone formation markers alkaline phosphatase (ALP) was 378 U/L and procollagen type-1 N-terminal propeptide (P1NP) was 1,092 μg/L, whereas the bone resorption markers type-I collagen cross-linked C-telopeptides (CTX) was 5.87 μg/L and tartrate-resistant acid phosphatase 5b (TRAP-5b) was 10.69 IU/L. Bone mineral density (BMD) was assessed by dual-energy X-ray absorptiometry (DXA). The BMD values were 0.692 g/cm^2^ in the lumbar spine (L1-L4), 0.721 g/cm^2^ in the femoral neck, and 0.802 g/cm^2^ in the total hip, with corresponding T-scores of −3.5, −2.0, and −1.8 at these respective anatomical sites. The patient’s coronary CT and chest CT scans revealed coronary artery calcification and extensive multifocal calcifications along the entire aorta. Fortunately, the patient had never experienced a low-energy fracture. This accurate preoperative assessment provided the patient with the confidence and courage to undergo a second operation. Subsequently, thoracoscopic ectopic parathyroidectomy was performed, and intraoperative parathyroid hormone monitoring (IOPTH) demonstrated a marked decline from 2,703 to −225 pg./mL within 5 min after excision. Pathological examination confirmed the presence of parathyroid adenoma with hyperplasia (Figure 3). Another postoperative complication was hungry bone syndrome. Cinacalcet was stopped preoperatively. However, a severe decrease in serum total calcium concentration and iPTH levels was still observed postoperatively, accompanied by hypophosphatemia, and lasted for approximately 7 days. The patient’s minimum serum total calcium level was 1.5 mmol/L, and the lowest iPTH level was 7.85 pg./mL. Despite the absence of apparent clinical signs of hypocalcemia, aggressive calcium supplementation was administered to shorten the duration of hungry bone syndrome. We switched the patient to peritoneal dialysate with a calcium concentration of 1.75 mmol/L and prescribed 4–6 g/day of oral and intravenous elemental calcium as well as 2 μg/day of calcitriol during the postoperative period. Intravenous calcium supplementation was discontinued from the seventh day after surgery when serum calcium levels increased to more than 2.1 mmol/L. The patient recovered well, with stable and normal biochemical parameters observed at both the second week (serum calcium: 2.16 mmol/L, iPTH: 65.6 pmol/L) and the sixth month following resection (serum calcium: 2.21 mmol/L, iPTH: 58.6 pmol/L). The patient underwent regular follow-up for 24 months postoperatively. During follow-up, the patient’s symptoms of bone pain and skin itching gradually resolved, and the associated psychological distress was completely alleviated. At the 2-year postoperative evaluation, bone turnover markers showed remarkable improvement: PTH at 98.7 pmol/L, serum calcium at 2.15 mmol/L, serum phosphorus at 1.27 mmol/L, ALP at 145 U/L, P1NP at 189 μg/L, CTX at 1.35 μg/L, and TRAP-5b at 4.35 IU/L, with all parameters returning to normal or near-normal ranges. Furthermore, DXA scans confirmed significant improvements in both bone mineral density and T-scores at the lumbar spine (L1–L4), femoral neck, and total hip compared with the preoperative baseline. All follow-up results remained stable, effectively ruling out the possibility of chronic hypoparathyroidism.

## Discussion

Ectopic parathyroid glands result from aberrant migration of the parathyroids during the early stages of embryonic development (5). The parathyroid glands originate from the endoderm and develop from the dorsal wings of the third and fourth pharyngeal pouches. Ectopic parathyroid glands can be located anywhere from the base of the tongue to the mediastinum. The superior parathyroid glands develop from the fourth pharyngeal pouch and are usually located at the posterior aspect of the cricothyroid junction. They are frequently found in the tracheoesophageal groove, retroesophageal space, posterior superior mediastinum, paraesophageal region, intrathyroidal location, or carotid sheath. The inferior parathyroid glands derive from the third pharyngeal pouch and follow the descending route of the thyroid gland along with the thymus. Thus, they are more frequently ectopic than the superior glands and are often found in the thymus, mediastinum, thyroid, thyrothymic ligament, and submandibular gland (7). In our case, only three hyperplastic parathyroid glands were identified in the thyroid gland region during the initial surgery, with two foci located on the right side (upper and lower poles) and one focus in the left parathyroid gland. Therefore, we infer that the ectopic parathyroid gland in the anterior mediastinum may have originated from the inferior parathyroid gland. Previous studies have reported that 6.6–26% of patients with renal HPT have parathyroid glands in ectopic locations (6, 7), which is higher than the rate generally reported in surgical series among patients with primary hyperparathyroidism (PHPT) (6–14%) (7, 8). It has also been reported that the incidence of ectopic parathyroid glands is higher in patients undergoing maintenance hemodialysis (7.4%) than in those in the predialysis stage (3.9%) (9). Ectopic parathyroid glands in renal HPT are associated with larger gland size, which may contribute to their descent from a normal anatomical position into an ectopic location (5).

Accurate localization of parathyroid glands is crucial for identifying ectopic parathyroid glands, thereby shortening operative time and reducing the risk of persistent and recurrent hyperparathyroidism after PTX. Preoperative imaging can aid in identifying parathyromatosis and supernumerary glands. However, there is no commonly accepted consensus for preoperative localization, as different imaging modalities (ultrasonography, Technetium-99 m methoxyisobutylisonitrile single photon emission computed tomography/computed tomography [^99m^Tc-MIBI SPECT/CT], and four-dimensional computed tomography [4D-CT]) have varying advantages and disadvantages (10). A meta-analysis reported that ultrasonography has a sensitivity of 76.1% and a positive predictive value of 93.2%, while ^99m^Tc-MIBI SPECT/CT scanning has a sensitivity of 78.9% and a positive predictive value of 90.7% (11). Therefore, the first-line modalities for reoperation localization of parathyroid lesions at our institution are ultrasonography and ^99m^Tc-MIBI SPECT/CT. When results from these two modalities are negative or discordant, 4D-CT or MRI is considered as a further option. Ultrasonography is a non-invasive, fast, and inexpensive imaging modality that does not involve ionizing radiation or contrast administration (12). Nonetheless, ultrasonography is operator-dependent and unable to visualize parathyroid adenomas within the mediastinum or in retroesophageal and retropharyngeal locations (10, 12, 13). ^99m^Tc-MIBI SPECT/CT is particularly useful for localizing ectopic glands (12). It provides both anatomic and functional information, enhancing the detection of ectopic glands or deep posterior lesions, which are easily overlooked by ultrasonography. This technique also reveals uptake foci and has a superior positive predictive value (83.5–96.0%) (14). In addition to the aforementioned imaging modalities, the intraoperative parathyroid hormone (IOPTH) protocol may be a valuable adjunct in predicting successful surgical outcomes. Previous studies have confirmed that the use of IOPTH is associated with higher cure rates and lower reoperation rates in patients with primary HPT (15). Although the value of IOPTH in parathyroid surgery for SHPT has not been conclusively confirmed, many researchers have attempted to establish a valid IOPTH protocol in patients with renal HPT. Several studies have predetermined a cut-off for IOPTH decline from baseline, ranging from 70 to 90% to predict successful surgery with a lower probability of SHPT recurrence and improved quality of life (16–19). In this case, ultrasonography and ^99m^Tc-MIBI SPECT/CT were performed prior to the initial surgery, revealing a mediastinal nodule that likely originated from the parathyroid gland. However, this nodule was not removed due to its weak function and the high risk associated with the operation. IOPTH monitoring was not performed during the initial surgery. The Japanese Society for Dialysis Therapy recommends a target range of iPTH after parathyroidectomy in patients with renal HPT of 60–240 pg./mL (20). In this case, iPTH decreased from 1,114 pg./mL to 303 pg./mL after the initial surgery, which did not reach the recommended range. However, the iPTH reduction was significantly higher than the minimum cut-off value of 70% for predicting surgical success, as mentioned above. Therefore, it is challenging to predict the recurrence of SHPT after initial surgery based on the current complex evidence. If IOPTH monitoring had been implemented, it might have provided a better prognostic prediction for this patient’s parathyroidectomy outcome. Nevertheless, this case offers valuable clinical insights that underscore the critical importance of routine IOPTH monitoring in parathyroid surgery.

In our hospital, precise localization using ^99m^Tc-MIBI SPECT/CT imaging and advanced thoracoscopic surgical techniques contributes to successful operations. This case illustrates that precise preoperative lesion localization and effective patient communication facilitate collaborative clinical decision-making, resulting in improved treatment efficacy and higher patient satisfaction. However, another challenge we must address is hungry bone syndrome (HBS). HBS is a notable potential complication after parathyroidectomy, characterized by rapid, profound (serum calcium < 2.1 mmol/L), and prolonged (lasting more than 4 days postoperatively) hypocalcemia (21). The prevalence of HBS varies from 15 to 92% in SHPT patients after PTX (21, 22). Risk factors for HBS include longer pre-surgery dialysis duration, younger age, higher body weight, higher preoperative alkaline phosphatase levels, greater weight of resected parathyroid glands, and lower preoperative calcium levels (22–29). Severe hypocalcemia is considered to be associated with the sudden reduction in serum iPTH concentration after PTX, which inhibits bone resorption and promotes bone formation, thereby accelerating skeletal calcium uptake. Severe hypocalcemia is considered to be associated with the sudden reduction in serum iPTH concentration after PTX, which inhibits bone resorption and promotes bone formation, thereby accelerating skeletal calcium uptake. Intravenous calcium supplementation is recommended to maintain normal blood calcium levels immediately after PTX. The National Kidney Foundation Kidney Disease Outcomes Quality Initiative (NKF KDOQI) recommends initiating oral calcium supplementation, such as, 1 to 2 g of calcium carbonate three times a day (for a total of 1.2–2.4 g/day of elemental calcium), when oral intake is possible (30). However, higher supplementation requirements may be encountered in clinical practice. According to previous studies, the average requirement for elemental calcium 1 week after surgery was 3.2 g/day (31), which was higher in HBS patients (8.5 g/day) (28). The most significant daily dose of elemental calcium ever reported was 35.9 g in an HBS patient after PTX (32). Adequate active vitamin D is necessary to maintain calcium levels. KDOQI recommends that calcitriol supplementation up to 2 μg/day, both postoperatively and preoperatively, may help maintain postoperative calcium levels (30). However, other studies have reported that the use of active vitamin D preoperatively was not significantly associated with a lower risk of HBS (28, 33, 34). Therefore, the role of active vitamin D in preventing HBS in SHPT patients remains uncertain.

Our patient differs from those undergoing maintenance hemodialysis. For patients undergoing PD, solute transport across the peritoneum occurs via convection or diffusion along a favorable concentration gradient. Since calcium absorption takes place by diffusion from the dialysate into the vascular compartment, using dialysate with 1.75 mmol/L calcium is considered effective (35). Previous studies have suggested that a dialysate calcium concentration of 1.75 mmol/L is superior to 1.25 mmol/L in increasing serum total calcium levels among PD patients (36, 37). Yang et al. reported that PD can alleviate the clinical course of HBS after PTX through the use of dialysate containing 1.50 mmol/L of calcium, reducing postoperative calcium supplementation and shortening hospitalization compared with hemodialysis in patients with SHPT. This effect may be attributed to stronger calcium replenishment from longer average daily dialysis time (38). Therefore, we believe that peritoneal dialysate with a higher calcium concentration may be a better option for shortening the course of HBS in patients undergoing PD.

According to previous research, the widespread use of calcimimetics could increase the incidence of hypocalcemia in patients on dialysis (39, 40). Therefore, cinacalcet was discontinued preoperatively in this case to reduce the risk of hypocalcemia. The patient received dialysate with a calcium concentration of 1.75 mmol/L, combined with adequate calcium and active vitamin D supplementation, which resulted in remission of HBS within 10 days after PTX.

With the advancement of medical research, treatment strategies for bone diseases in dialysis patients have continued to make progress. We plan to regularly monitor the patient’s bone turnover status to guide optimal treatment selection. If a high bone turnover status occurs, it is preferable to select antiresorptive medications, including bisphosphonates, receptor activator of nuclear factor κB ligand (RANKL) inhibitors, and hormone therapies, to reduce bone turnover. If the patient presents with a low bone turnover state, PTH analogues that stimulate bone turnover, such as teriparatide and abaloparatide, could be a better choice (41).

## Conclusion

PTX is a reasonable option for patients with ESKD complicated by medically refractory SHPT. Our case suggests that the combination of ^99m^Tc-MIBI SPECT/CT and ultrasonography is useful for the precise localization of the parathyroid glands, especially for identifying ectopic parathyroid glands. IOPTH monitoring is an alternative method to increase the likelihood of surgical success and reduce the recurrence of SHPT. The incidence of HBS is relatively higher in patients with renal HPT. Reasonable individualized postoperative management is necessary for PD patients undergoing PTX, including adequate calcium and active vitamin D supplementation, as well as the use of peritoneal dialysate with a higher calcium concentration.

## Data Availability

The original contributions presented in the study are included in the article/supplementary material, further inquiries can be directed to the corresponding author.
